# Short-term clinical outcomes associated with a standardized multicomponent facial rehabilitation program in acute or early-subacute peripheral facial nerve palsy: a prospective controlled cohort study

**DOI:** 10.3389/fneur.2026.1931200

**Published:** 2026-09-23

**Authors:** Wong Guo Lin

**Affiliations:** School of Traditional Chinese Medicine of Xiamen University Malaysia, Sepang, Selangor, Malaysia

**Keywords:** acupuncture, Bell’s palsy, facial exercise, facial massage, peripheral facial nerve palsy, rehabilitation

## Abstract

**Background:**

Peripheral facial nerve palsy can impair facial symmetry, eye closure, oral competence, communication, and quality of life. Although spontaneous recovery is common, some patients experience persistent functional and psychosocial impairment. Evidence for structured adjunctive rehabilitation during the acute and early-subacute phases remains limited. We examined whether a standardized multicomponent facial rehabilitation program, added to usual care, was associated with more favorable short-term outcomes than usual care alone.

**Methods:**

This prospective controlled cohort study included 160 adults with acute or early-subacute unilateral peripheral facial nerve palsy. Eighty-four participants received usual care plus a standardized program comprising facial acupuncture, therapist-assisted facial massage, supervised facial movement training, and daily home exercises over 12 supervised sessions during 6 weeks; 76 received usual care alone. Allocation was non-randomized and based on clinician recommendation and patient preference, and outcome assessors were not blinded. The primary outcome was Week 12 Sunnybrook Facial Grading System (SFGS) total score. Secondary outcomes included SFGS subscales, House–Brackmann outcomes, Facial Disability Index (FDI), Facial Clinimetric Evaluation (FaCE), pain, and stiffness. Adjusted between-group differences were estimated using multivariable regression controlling for baseline values and prespecified clinical covariates.

**Results:**

SFGS scores improved in both groups, with greater observed improvement in participants receiving the multicomponent program. At Week 12, mean SFGS scores were 82.5 ± 11.8 versus 69.5 ± 11.5, with mean improvements of 46.7 ± 12.6 versus 33.2 ± 12.5 points. The adjusted between-group difference was 12.7 points (95% CI, 8.8–16.7; *p* < 0.001). Repeated-measures analysis showed a significant group-by-time interaction (*p* < 0.001). More favorable observed outcomes were also found for FDI physical and social/wellbeing scores, FaCE total score, stiffness, and House–Brackmann recovery thresholds. House–Brackmann Grade I–II was achieved by 79.8% versus 34.2% at Week 12.

**Conclusion:**

The multicomponent rehabilitation program was associated with more favorable short-term clinician-rated and patient-reported outcomes. Given the non-randomized allocation and unblinded assessment, these findings are hypothesis-generating and require confirmation in randomized, blinded, attention-matched trials.

## Introduction

Peripheral facial nerve palsy is a common and clinically consequential disorder that disrupts facial expression, eye closure, oral competence, speech, eating, and social communication. Bell’s palsy is the most frequent cause of acute unilateral peripheral facial nerve palsy and has an estimated annual incidence of approximately 11.5–53.3 per 100,000 individuals across populations (1). Although many patients recover substantially, the course is variable, and delayed or incomplete recovery can leave patients with persistent asymmetry, stiffness, synkinesis, and reduced quality of life (2). Because facial movement is central to nonverbal communication and self-image, even residual deficits that appear moderate on clinician-rated scales may be highly disabling from the patient’s perspective.

Current evidence-based management of acute Bell’s palsy emphasizes timely diagnosis, eye protection, and early corticosteroid treatment. Clinical practice guidelines recommend oral corticosteroids within 72 h for appropriate patients and do not support antiviral monotherapy as routine treatment (3). In the landmark randomized trial by Sullivan and colleagues, prednisolone significantly improved complete recovery at 3 and 9 months, whereas acyclovir alone showed no clear benefit (4). However, pharmacologic treatment primarily targets the early inflammatory phase; it does not directly address facial motor relearning, resting asymmetry, stiffness, patient-perceived disability, or the development of maladaptive movement patterns during recovery. This creates a clinically relevant space for structured adjunctive rehabilitation strategies that are safe, reproducible, and feasible during the acute and early-subacute phases.

Accordingly, there has been growing interest in adjunctive therapeutic approaches aimed at enhancing recovery beyond standard pharmacologic management. These approaches encompass a range of supportive and rehabilitative strategies; for example, a recent case series by Singh et al. reported favorable outcomes with hyperbaric oxygen therapy as an adjunctive treatment for Bell’s palsy, although the preliminary nature of such evidence highlights the need for further controlled studies (5). Within this broader context, structured facial rehabilitation and acupuncture-assisted interventions have also attracted increasing clinical interest.

The rehabilitation literature for facial palsy has grown, but important uncertainties remain. A Cochrane review concluded that high-quality evidence for physical therapy in idiopathic facial paralysis was limited, although tailored facial exercises may improve facial function in moderate paralysis and chronic cases and may reduce sequelae in acute cases (6). An updated systematic review similarly identified facial exercise therapy as the most widely evaluated rehabilitation approach, while emphasizing heterogeneity in interventions, outcome measures, and study quality (7). Recent randomized trials have provided encouraging signals: early physical therapy may be particularly helpful in severe Bell’s palsy (8), mirror-based rehabilitation initiated in the acute phase has shown promising long-term results (9), and structured facial stretching with exercise improved Sunnybrook Facial Grading System (SFGS) scores more than conventional exercise in acute Bell’s palsy (10). Nevertheless, the field still lacks pragmatic evidence on standardized combined protocols that integrate supervised facial rehabilitation with manual therapy and acupuncture-assisted treatment across serial early recovery time points.

Acupuncture is widely used for Bell’s palsy, particularly in East Asian clinical practice, but its role remains debated because of heterogeneity and limitations in prior trials. A systematic review and meta-analysis suggested a possible benefit, but concluded that evidence for efficacy and safety was insufficient because of poor study quality and heterogeneity (11). Individual studies suggest that acupuncture-related treatment characteristics and patient selection may matter: strengthened stimulation eliciting de qi improved facial nerve function in acute Bell’s palsy (12), while acupuncture improved physical and social disability in patients with Bell’s palsy sequelae compared with a waiting-list control (13). However, many prior acupuncture studies have evaluated acupuncture as a discrete modality, in chronic sequelae, or with variable protocols, rather than as part of a clearly standardized combined rehabilitation package delivered during early recovery.

Another limitation of existing evidence is the frequent reliance on broad global grading systems or single endpoint assessments. The House–Brackmann grading system remains clinically familiar, but its ordinal global structure may be less sensitive to regional facial changes. The SFGS provides a more detailed assessment by separately evaluating resting symmetry, voluntary movement, and synkinesis, producing a 0–100 composite score that is well suited for measuring change over time (14, 15). Patient-reported instruments such as the Facial Disability Index (FDI) and Facial Clinimetric Evaluation (FaCE), further capture physical, social, and quality-of-life consequences that may not be fully reflected by clinician-rated facial movement alone (16, 17). Therefore, studies that combine serial SFGS assessment with patient-reported outcomes may better characterize both the speed and clinical meaning of recovery.

The present study was designed to evaluate a standardized multicomponent facial rehabilitation program comprising facial acupuncture, therapist-assisted massage, supervised facial movement training, and home exercise in adults with acute or early-subacute unilateral peripheral facial nerve palsy using a prospective controlled cohort design. The study incorporated serial assessments from baseline to Week 12, clinician-rated outcomes, patient-reported functional and quality-of-life measures, and symptom outcomes. The primary aim was to determine whether receiving the standardized protocol in addition to usual care was associated with a greater Week 12 SFGS total score compared with usual care/control management. Secondary aims were to compare recovery trajectories, SFGS subscale changes, House–Brackmann recovery thresholds, FDI physical and social/wellbeing scores, pain, and stiffness. It was hypothesized that the standardized combined protocol would be associated with faster and greater improvement in facial motor function and patient-perceived recovery over 12 weeks.

## Methods

### Study design and reporting framework

This study was designed as a prospective controlled cohort study examining clinical outcomes associated with receipt of a standardized multicomponent facial rehabilitation program in patients with acute or early-subacute unilateral peripheral facial nerve palsy. The program comprised facial acupuncture, therapist-assisted facial massage, supervised facial movement training, and a daily home exercise program. Participants who received the combined program in addition to usual care were compared with a contemporaneous group receiving usual care without the structured program. Because treatment assignment was not randomized, group status was considered an observational exposure rather than a randomized treatment allocation. The study is reported in accordance with the Strengthening the Reporting of Observational Studies in Epidemiology (STROBE) guideline for cohort studies.

### Setting

Participants were recruited from patients who were referred to the school of Traditional Chinese Medicine between January 2023 and January 2026. Baseline assessment was performed before initiation of the intervention protocol. Follow-up assessments were scheduled at baseline, Week 2, Week 4, Week 8, and Week 12.

### Participants

Eligible participants were (1) adults aged ≥18 years, (2) with clinically diagnosed unilateral peripheral facial nerve palsy, including Bell’s palsy and post-infectious peripheral facial palsy, (3) who were referred during the acute or early-subacute phase of disease within 14 days from symptom onset to baseline assessment. All participants were able to complete clinical patient-reported questionnaires. Written informed consent was completed by the participants before their recruitment. Patients were excluded if they had central facial palsy, bilateral facial palsy, recurrent facial palsy, traumatic or postsurgical facial nerve injury, Ramsay Hunt syndrome with active vesicular disease, progressive neurological deficits, suspected stroke or central nervous system disease, prior acupuncture or structured facial rehabilitation for the same episode before baseline, contraindications to acupuncture or facial massage, active infection or skin lesions at treatment sites, or inability to complete follow-up assessments.

The diagnosis of peripheral facial nerve palsy was established by a neurologist based on the clinical history and physical and neurological examination. Bell’s palsy was operationally defined as an acute unilateral lower-motor-neuron facial nerve palsy for which no alternative cause was identified after clinical assessment. Features suggesting central facial weakness, bilateral or recurrent palsy, traumatic or postsurgical nerve injury, active Ramsay Hunt syndrome, progressive neurological disease, or another identifiable structural cause were considered inconsistent with Bell’s palsy. Post-infectious peripheral facial palsy was defined as acute unilateral lower-motor-neuron facial weakness occurring in temporal association with an infection, in the absence of evidence for a central, traumatic, postsurgical, or other defined cause. Imaging, electrophysiological studies, or laboratory investigations were performed selectively according to clinical indication.

### Group allocation

Participants were classified according to the care received during follow-up. The multicomponent rehabilitation group received usual care plus the standardized combined program comprising facial acupuncture, therapist-assisted facial massage, supervised facial movement training, and a daily home exercise program. The usual-care group received usual care without this structured program. Allocation was not randomized and was influenced by clinician recommendation and patient preference. Outcome assessors were aware of group assignment. Accordingly, the observed between-group differences represent observational associations and may be influenced by measured and unmeasured differences between groups. Prespecified baseline characteristics were incorporated into adjusted analyses to reduce measured confounding; however, such adjustment cannot eliminate residual confounding, treatment-preference effects, expectation or contextual effects, or differences in therapeutic attention.

### Multicomponent rehabilitation program

The multicomponent rehabilitation program consisted of facial acupuncture, therapist-assisted facial massage, supervised facial movement training, and a daily home exercise program delivered over 6 weeks, with two supervised sessions per week for a total of 12 sessions. Each session was performed by a licensed acupuncturist using a predefined treatment sequence. The protocol included facial acupuncture, gentle therapist-assisted facial massage, supervised facial movement training, and a home exercise program. Acupuncture was performed using sterile disposable needles at predefined facial and distal acupuncture points selected for peripheral facial palsy rehabilitation (Figure 1). Facial massage consisted of gentle soft-tissue mobilization and directional massage of the forehead, peri-orbital, cheek, perioral, and mandibular regions, avoiding excessive force or painful stretching. Supervised exercises included mirror-guided eyebrow elevation, gentle eye closure, smiling, teeth showing/snarl movement, lip puckering, cheek inflation, and controlled relaxation between movements. Participants were instructed to perform the home exercise program daily for approximately 10 min/day (Figure 2). Clinician-rated facial outcomes, including the SFGS and House–Brackmann grade, were assessed in person by a single clinician experienced in the administration and grading of both instruments. The same assessor performed the clinical ratings throughout the study period and was aware of participants’ group allocation.

**Figure 1:**
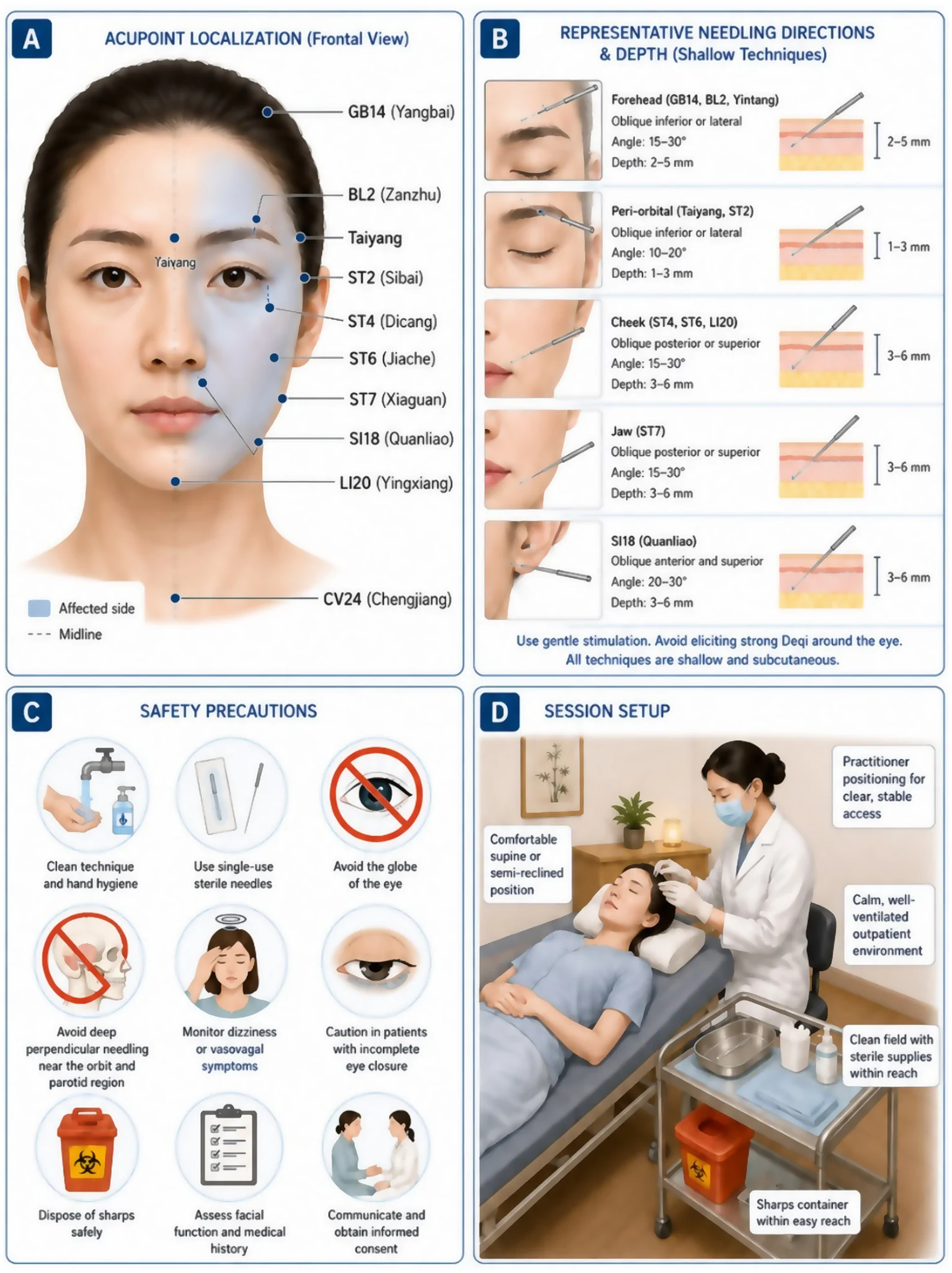
Acupuncture protocol guide for peripheral facial nerve palsy. This figure provides a standardized visual guide for acupuncture treatment in patients with peripheral facial nerve palsy. **(A)** illustrates the frontal localization of used facial acupoints on the affected side, including GB14 (Yangbai), BL2 (Zanzhu), Taiyang, ST2 (Sibai), ST4 (Dicang), ST6 (Jiache), ST7 (Xiaguan), SI18 (Quanliao), LI20 (Yingxiang), Yintang, and CV24 (Chengjiang). The affected side and facial midline are indicated to support consistent point identification. **(B)** summarizes representative shallow needling directions and approximate depths for major anatomical regions, including the forehead, peri-orbital region, cheek, jaw, and SI18 area. Oblique, superficial, and subcutaneous techniques are emphasized, with reduced depth around the peri-orbital region to minimize ocular risk. **(C)** highlights key safety precautions, including clean technique, use of single-use sterile needles, avoidance of the globe of the eye, avoidance of deep perpendicular needling near the orbit and parotid region, monitoring for dizziness or vasovagal symptoms, caution in patients with incomplete eye closure, safe disposal of sharps, assessment of facial function and medical history, and informed consent. **(D)** demonstrates the recommended outpatient treatment setup, including patient positioning, practitioner access, sterile field preparation, availability of sterile supplies, calm and well-ventilated treatment conditions, and appropriate placement of a sharps container.

**Figure 2:**
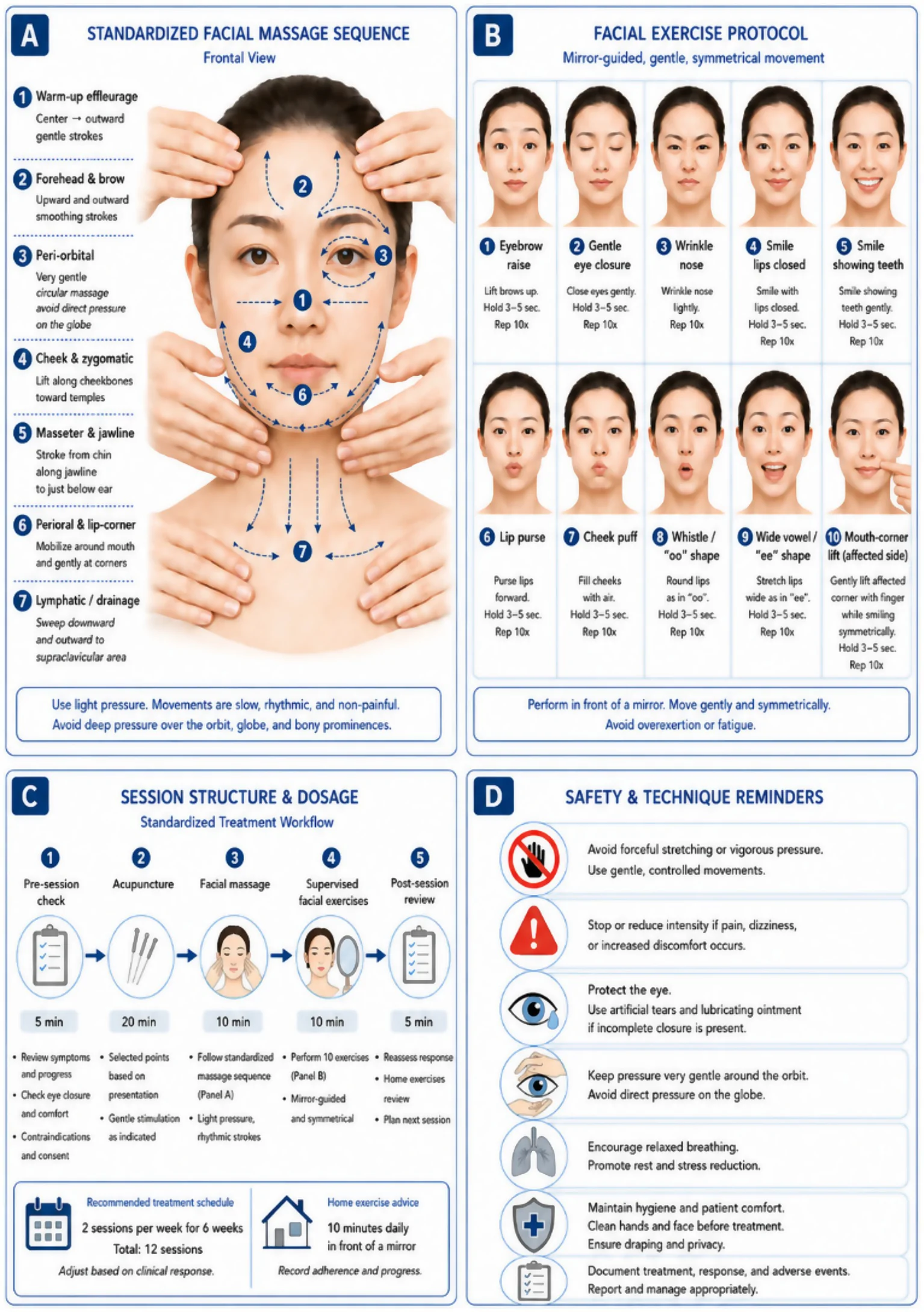
Standardized adjunctive facial rehabilitation protocol for peripheral facial nerve palsy. This figure summarizes the non-needling components of the treatment protocol, including facial massage, facial exercise, session dosage, and safety reminders. **(A)** presents a standardized facial massage sequence performed using light, slow, rhythmic, and non-painful pressure. The sequence includes warm-up effleurage, forehead and brow smoothing, very gentle peri-orbital circular massage, cheek and zygomatic lifting strokes, masseter and jawline strokes, perioral and lip-corner mobilization, and lymphatic drainage toward the supraclavicular region. Direct pressure over the globe, orbit, and bony prominences should be avoided. **(B)** illustrates a mirror-guided facial exercise protocol designed to encourage gentle and symmetrical facial movement. The exercises include eyebrow raise, gentle eye closure, nose wrinkle, closed-lip smile, teeth-showing smile, lip purse, cheek puff, whistle/“oo” shape, wide vowel/“ee” shape, and assisted mouth-corner lift on the affected side. Each exercise is performed gently, typically held for 3–5 s and repeated 10 times, while avoiding fatigue or overexertion. **(C)** outlines the standardized treatment workflow and dosage, including pre-session assessment, acupuncture, facial massage, supervised facial exercises, and post-session review. The proposed schedule consists of two sessions per week for 6 weeks, with daily home facial exercises performed in front of a mirror. **(D)** summarizes safety and technique reminders, including avoidance of forceful stretching, reduction of intensity if pain or dizziness occurs, eye protection in patients with incomplete closure, gentle peri-orbital pressure, relaxed breathing, hygiene and patient comfort, and documentation of treatment response and adverse events. This rehabilitation sequence is intended to complement acupuncture and to promote safe, structured, and reproducible facial recovery training.

### Comparator

The usual-care group received usual care according to local clinical practice. Usual care included corticosteroids, antiviral therapy when clinically indicated, eye protection, lubricating eye drops, taping or eye-care advice, general facial movement advice, analgesics if needed, and routine follow-up. Use of corticosteroids and timing of steroid initiation were recorded. The usual-care group did not receive the standardized multicomponent rehabilitation program during the study period.

## Outcomes

### Primary outcome

The primary outcome was the SFGS total score at Week 12, analyzed with adjustment for baseline total score and prespecified clinical covariates. The SFGS total score ranges from 0 to 100, with higher scores indicating better facial function. Change from baseline to Week 12 was also reported.

### Sunnybrook subscale outcomes

SFGS subscale outcomes included voluntary movement, resting symmetry, and synkinesis scores. Voluntary movement scores indicate active facial movement, with higher scores reflecting better function. Resting symmetry and synkinesis scores are impairment scores, with lower values indicating better status. Subscale trajectories were summarized from baseline through Week 12.

### House–Brackmann outcomes

Facial nerve function was also assessed using the House–Brackmann grading system. Grades range from I to VI, where Grade I indicates normal facial function and Grade VI indicates complete paralysis. House–Brackmann outcomes included Week 12 grade, grade improvement from baseline to Week 12, ≥1-grade improvement, ≥2-grade improvement, and achievement of Grade I–II at Week 12.

### Patient-reported outcomes

The FDI was used to assess patient-reported physical and social/wellbeing function. FDI physical function and FDI social/wellbeing scores range from 0 to 100, with higher scores indicating better function or wellbeing. The Facial Clinimetric Evaluation total score was used as an additional facial-palsy-specific quality-of-life measure. FaCE total score ranges from 0 to 100, with higher scores indicating better facial-palsy-related quality of life.

### Symptom outcomes

Pain and facial stiffness/tightness were assessed using separate 0–10 numeric rating scales. For both scales, 0 indicated no symptom, and 10 indicated the worst imaginable symptom severity. Lower Week 12 scores and negative change scores indicated improvement.

### Covariates and potential confounders

Prespecified baseline covariates included age, sex, time from symptom onset to baseline assessment, baseline House–Brackmann grade, baseline Sunnybrook total score, diabetes mellitus, steroid start day from symptom onset, diagnosis type, and hypertension. The primary adjusted model included baseline SFGS, time from symptom onset to baseline, baseline House–Brackmann grade, age, sex, and diabetes. These variables were selected because they are clinically relevant predictors of recovery and potential confounders of the relationship between treatment group and outcome.

### Study size

A total of 182 patients were assessed for eligibility. Of these, 19 were excluded because they declined to participate in the study or they presented during the chronic phase and did not meet the prespecified eligibility criteria. The remaining 163 participants entered the study, including 85 in the intervention group and 78 in the control group. During follow-up, one participant in the intervention group and two participants in the control group were lost to follow-up. Consequently, the final analytic cohort comprised 160 participants, including 84 in the intervention group and 76 in the control group. No additional outcome data were missing among participants included in the final analysis (Figure 3). We included 160 participants, including 84 in the intervention group and 76 in the control group. The sample size was based on the number of eligible patients available during the study period. The achieved sample size was considered adequate for estimating adjusted between-group differences in SFGS recovery trajectories and for conducting exploratory subgroup and sensitivity analyses.

**Figure 3:**
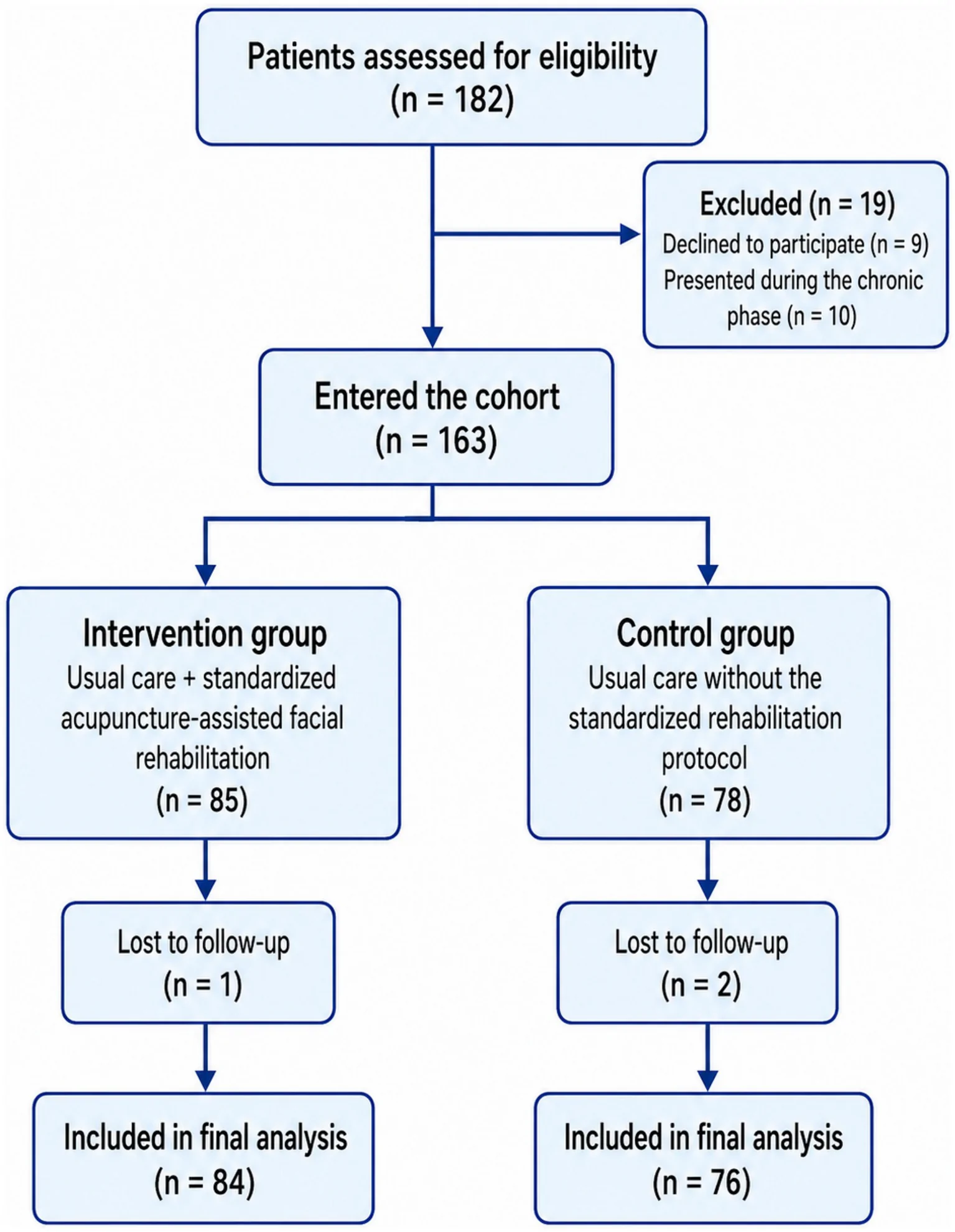
Participant flow through screening, enrollment, follow-up, and final analysis. A total of 182 patients were assessed for eligibility, of whom 19 were excluded because they declined to participate in this study or they presented during the chronic phase. The remaining 163 participants entered the study, including 85 in the intervention group and 78 in the control group. During follow-up, one participant in the intervention group and two participants in the control group were lost to follow-up. The final analytic cohort therefore comprised 160 participants: 84 in the intervention group and 76 in the control group.

### Statistical analysis

Continuous variables were summarized as mean ± standard deviation when normally distributed and as median with interquartile range when ordinal or skewed. Categorical variables were summarized as frequencies and percentages. Baseline characteristics were compared descriptively between treatment groups. Welch’s independent-samples *t*-test was used for continuous variables summarized as mean ± standard deviation. The Mann–Whitney *U* test was used for ordinal or skewed variables summarized as median with interquartile range. Fisher’s exact test was used for binary categorical variables, and Pearson’s *χ*^2^ test was used for multi-category categorical variables. The primary analysis estimated the adjusted between-group difference in the Week 12 SFGS total score using multivariable linear regression. The dependent variable was the Week 12 SFGS total score, and the main independent variable was treatment group. The model adjusted for baseline SFGS total score, time from symptom onset to baseline, baseline House–Brackmann grade, age, sex, and diabetes mellitus. The adjusted effect estimate was reported as the mean difference between intervention and control groups with 95% confidence interval and *p*-value. SFGS total score trajectory from baseline to Week 12 was further evaluated using a repeated-measures generalized estimating equation model. The model included treatment group, time point, and a group-by-time interaction term, with time treated as a categorical variable. An exchangeable working correlation structure was used, and robust standard errors were applied. The global group-by-time interaction was used to assess whether the recovery trajectory differed between groups over time.

Secondary continuous outcomes at Week 12 were analyzed using separate multivariable linear regression models. For each outcome, the Week 12 value was modeled as the dependent variable, and the treatment group was the main independent variable. Models were adjusted for the corresponding baseline outcome value, time from symptom onset to baseline, baseline House–Brackmann grade, age, sex, and diabetes mellitus. This approach was used for FDI physical function, FDI social/wellbeing, FaCE total score, pain score, and stiffness score. For FDI and FaCE outcomes, positive adjusted differences favored the intervention. For pain and stiffness NRS outcomes, negative adjusted differences favored the intervention. House–Brackmann baseline and Week 12 grades were summarized as mean ± standard deviation. Adjusted Week 12 House–Brackmann grade was also analyzed using linear regression adjusted for baseline House–Brackmann grade, baseline SFGS total score, time from symptom onset to baseline, age, sex, and diabetes. Binary House–Brackmann recovery outcomes, including ≥1-grade improvement, ≥2-grade improvement, and Week 12 Grade I–II status, were also summarized as *n* (%) and compared using absolute risk differences with 95% confidence intervals and Fisher’s exact test.

Exploratory subgroup analyses were performed for the primary Week 12 SFGS total score outcome. Subgroups included onset-to-baseline interval (≤7 days vs. 8–14 days), baseline severity (House–Brackmann III–IV vs. V–VI), age (<45 vs. ≥ 45 years), sex, and diagnosis type. Within each subgroup level, adjusted between-group differences were estimated using linear regression adjusted for the prespecified covariates, excluding the subgroup-defining variable when applicable. Treatment-effect modification was assessed using interaction terms between treatment group and subgroup indicator. These analyses were considered exploratory and were not adjusted for multiplicity.

Sensitivity analyses assessed the numerical stability of the primary between-group association across alternative analytic specification. These included an unadjusted Week 12 score difference, an unadjusted change-score comparison, a baseline-adjusted ANCOVA model, a parsimonious adjusted model including baseline SFGS total score, onset-to-baseline time, and baseline House–Brackmann grade, the primary adjusted model, an expanded covariate model additionally including steroid start day, diagnosis type, and hypertension, propensity-score weighted analysis using stabilized inverse-probability weights, robust regression using Huber M-estimation, median regression using quantile regression, and repeated-measures GEE analysis across all Sunnybrook time points.

All statistical tests were two-sided. A *p*-value <0.05 was considered statistically significant for the primary analysis. Because multiple secondary, subgroup, and sensitivity analyses were performed, these findings were interpreted as supportive or exploratory evidence.

Statistical significance and clinical importance were considered separately. No minimal clinically important difference (MCID) for the SFGS was prespecified in the original cohort design. For clinical interpretation of the present results, a 10-point SFGS difference was used *post hoc* as an externally supported benchmark based on a recent evidence-based clinical practice guideline for facial nerve palsy (18). This benchmark was used descriptively and was not treated as an additional hypothesis-testing threshold. Published minimal important change estimates for patient-reported facial-palsy instruments were considered only as contextual information because they were derived in populations differing from the present acute/early-subacute cohort. In the absence of well-established population-specific thresholds, statistical significance alone was not considered sufficient to establish the clinical importance of symptom outcomes.

### Bias and confounding control

Several design and analytic steps were used to reduce potential bias. Eligibility criteria were restricted to acute or early-subacute unilateral peripheral facial nerve palsy to improve clinical homogeneity. Baseline disease severity, symptom duration, steroid timing, and major comorbidities were recorded. The primary analysis adjusted for baseline SFGS score, time from symptom onset, baseline House–Brackmann grade, age, sex, and diabetes. Additional analyses using expanded covariate adjustment and propensity-score weighting were performed to assess the stability of the estimated between-group association to alternative approaches for controlling measured confounding. These methods can address only measured covariates and cannot eliminate bias related to treatment preference, patient expectations, placebo or contextual effects, differential therapeutic attention, motivation, adherence, or other unmeasured characteristics.

## Results

### Study population and baseline characteristics

A total of 160 patients with peripheral facial nerve palsy were included in the analysis, of whom 84 received the multicomponent rehabilitation program and 76 were included in the control group. Baseline demographic and clinical characteristics were broadly comparable between the two groups (Table 1). The mean age was 45.1 ± 12.6 years in the intervention group and 44.1 ± 12.5 years in the control group, and 41 patients (48.8%) and 32 patients (42.1%), respectively, were female. Median time from symptom onset to baseline assessment was 12 (9, 14) days in the intervention group and 11 (9, 14) days in the control group. Bell’s palsy was the most common diagnosis in both groups, accounting for 64 patients (76.2%) in the intervention group and 64 patients (84.2%) in the control group.

**Table 1:** Baseline demographic and clinical characteristics of the study cohort.

Characteristic	Intervention (*n* = 84)	Control (*n* = 76)	*p* value
Age, years	45.1 ± 12.6	44.1 ± 12.5	0.606
Female sex, *n* (%)	41 (48.8)	32 (42.1)	0.429
Height, cm	167.9 ± 10.6	168.3 ± 10.7	0.776
Weight, kg	69.7 ± 13.9	70.7 ± 13.9	0.643
BMI, kg/m^2^	25.1 ± 6.2	25.2 ± 5.6	0.918
Education level, *n* (%)			0.813
Primary or less	4 (4.8)	2 (2.6)	
Secondary	25 (29.8)	20 (26.3)	
College/university	36 (42.9)	37 (48.7)	
Postgraduate	19 (22.6)	17 (22.4)	
Bell’s palsy diagnosis, *n* (%)	64 (76.2)	64 (84.2)	0.238
Right-sided palsy, *n* (%)	49 (58.3)	40 (52.6)	0.525
Time from symptom onset to baseline, days	12 [9, 14]	11 [9, 14]	0.562
Symptom onset ≤7 days, *n* (%)	9 (10.7)	7 (9.2)	0.798
Steroid start from onset, days	4 [2, 5]	3 [2, 5]	0.327
Steroid started ≤3 days, *n* (%)	39 (46.4)	40 (52.6)	0.527
Diabetes mellitus, *n* (%)	7 (8.3)	3 (3.9)	0.334
Hypertension, *n* (%)	21 (25.0)	16 (21.1)	0.579
Former smoking, *n* (%)	11 (13.1)	5 (6.6)	0.196
Baseline House–Brackmann grade, *n* (%)			0.602
Grade 3	12 (14.3)	11 (14.5)	
Grade 4	62 (73.8)	57 (75.0)	
Grade 5	8 (9.5)	4 (5.3)	
Grade 6	2 (2.4)	4 (5.3)	
Sunnybrook total score	35.8 ± 3.7	36.3 ± 4.4	0.404
Sunnybrook voluntary movement score	51.3 ± 3.7	51.8 ± 4.3	0.384
Sunnybrook resting symmetry score	15.2 ± 0.9	15.1 ± 0.6	0.354
Sunnybrook synkinesis score	0 [0, 1]	0 [0, 1]	0.134
FDI physical function score	62.2 ± 21.1	61.6 ± 17.9	0.857
FDI social/wellbeing score	49.5 ± 16.4	51.6 ± 16.3	0.407
FaCE total score	48.1 ± 10.8	48.5 ± 15.5	0.864
Pain NRS score	3.1 ± 0.8	2.9 ± 0.9	0.132
Stiffness NRS score	4.3 ± 1.1	4.0 ± 1.0	0.147

Values are presented as mean ± SD, median [IQR], or *n* (%), as indicated. *p* values are descriptive and compare the intervention and control groups at baseline. Welch’s independent-samples *t* test was used for continuous variables summarized as mean ± SD; Mann–Whitney *U* test was used for ordinal or skewed variables summarized as median [IQR]; Fisher’s exact test was used for binary categorical variables; and Pearson’s *χ*^2^ test was used for multi-category categorical variables. BMI, body mass index; FaCE, Facial Clinimetric Evaluation; FDI, Facial Disability Index; IQR, interquartile range; NRS, numeric rating scale; SD, standard deviation.

Baseline facial nerve dysfunction was similar between groups. Baseline House–Brackmann grade was 4.0 ± 0.6 in both groups, and most patients had grade IV palsy at enrollment. Baseline SFGS total score was 35.8 ± 3.7 in the intervention group and 36.3 ± 4.4 in the control group. Baseline SFGS subscale scores, FDI physical and social/wellbeing scores, FaCE total score, pain NRS score, and stiffness NRS score were also comparable between groups (Table 1).

### Primary outcome: SFGS total score trajectory

SFGS total scores improved over time in both groups, with greater improvement in the intervention group across all follow-up time points (Table 2; Figure 4). At Week 2, the mean SFGS total score increased to 51.3 ± 5.7 in the intervention group and 43.7 ± 6.7 in the control group, corresponding to mean changes from baseline of 15.6 ± 7.0 and 7.4 ± 8.2 points, respectively. The adjusted between-group difference at Week 2 was 7.5 points (95% CI, 5.5–9.5; *p* < 0.001).

**Table 2:** Sunnybrook Facial Grading System total score trajectory from baseline to Week 12.

Time point	Intervention (*n* = 84)	Control (*n* = 76)	Intervention change from baseline	Control change from baseline	Adjusted between-group difference (95% CI)	*p* value
Baseline	35.8 ± 3.7	36.3 ± 4.4	–	–	–	0.404
Week 2	51.3 ± 5.7	43.7 ± 6.7	15.6 ± 7.0	7.4 ± 8.2	7.5 (5.5–9.5)	<0.001
Week 4	68.9 ± 8.7	53.5 ± 10.2	33.1 ± 10.0	17.1 ± 11.2	15.3 (12.2–18.4)	<0.001
Week 8	76.6 ± 14.0	63.9 ± 14.1	40.8 ± 14.1	27.6 ± 14.1	12.2 (7.6–16.8)	<0.001
Week 12	82.5 ± 11.8	69.5 ± 11.5	46.7 ± 12.6	33.2 ± 12.5	12.7 (8.8–16.7)	<0.001

Values are mean ± SD unless otherwise indicated. Changes are calculated as follow-up score minus baseline score. Adjusted between-group differences are intervention minus control and were estimated using linear regression for each follow-up time point, adjusted for baseline Sunnybrook total score, time from symptom onset to baseline, baseline House-Brackmann grade, age, sex, and diabetes. Heteroscedasticity-consistent (HC3) standard errors were used. The baseline *p* value was calculated using Welch’s independent-samples *t* test. Follow-up *p* values correspond to the adjusted between-group differences. A repeated-measures generalized estimating equation model including a group-by-time interaction showed a significant differential trajectory over time (overall group-by-time interaction *p* < 0.001). CI, confidence interval; GEE, generalized estimating equation; HB, House-Brackmann; SD, standard deviation; SFGS, Sunnybrook Facial Grading System.

**Figure 4:**
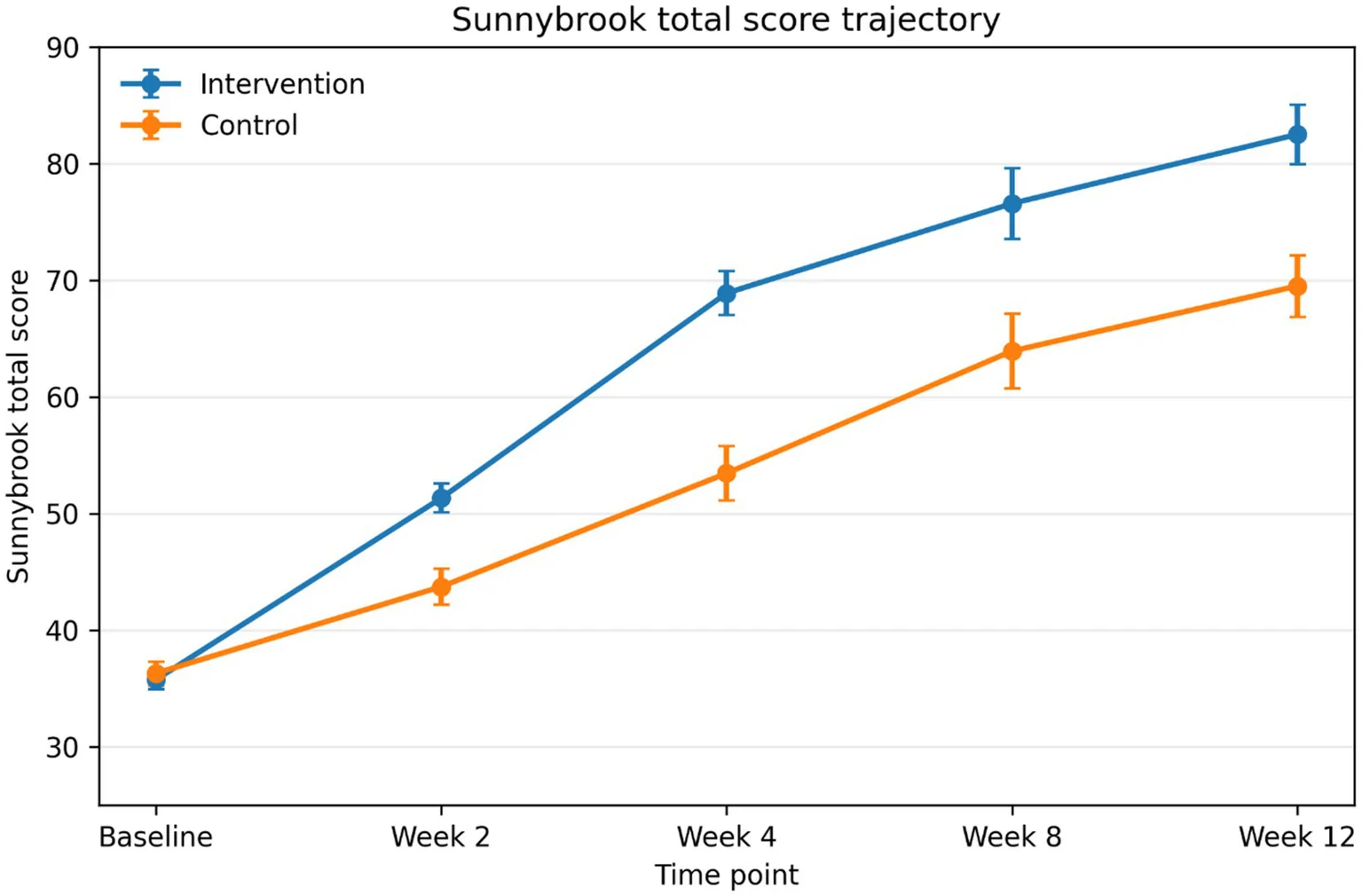
Sunnybrook Facial Grading System total score trajectory from baseline to Week 12. Values represent mean Sunnybrook total scores with 95% confidence intervals. Higher scores indicate better facial function. The intervention group showed greater observed improvement across follow-up.

The between-group difference remained evident at later follow-up. At Week 4, the intervention group had a mean SFGS total score of 68.9 ± 8.7 compared with 53.5 ± 10.2 in the control group, with an adjusted between-group difference of 15.3 points (95% CI, 12.2–18.4; *p* < 0.001). At Week 8, the corresponding scores were 76.6 ± 14.0 and 63.9 ± 14.1, respectively, with an adjusted difference of 12.2 points (95% CI, 7.6–16.8; *p* < 0.001). By Week 12, the mean SFGS total score was 82.5 ± 11.8 in the intervention group and 69.5 ± 11.5 in the control group. Mean change from baseline to Week 12 was 46.7 ± 12.6 points in the intervention group and 33.2 ± 12.5 points in the control group, with an adjusted between-group difference of 12.7 points (95% CI, 8.8–16.7; *p* < 0.001). A repeated-measures GEE model showed a significant group-by-time interaction for the SFGS total score trajectory (*p* < 0.001).

For clinical interpretation, the adjusted between-group differences were additionally considered against the externally supported 10-point SFGS benchmark. The Week 2 difference of 7.5 points (95% CI, 5.5–9.5) was below this benchmark despite statistical significance. At Week 4, the adjusted difference of 15.3 points (95% CI, 12.2–18.4) exceeded the benchmark across the entire confidence interval. At Weeks 8 and 12, the point estimates exceeded 10 points, but the corresponding confidence intervals included differences below this threshold. Thus, the later estimates were compatible with a clinically important difference but did not establish that the underlying between-group difference necessarily exceeded the external benchmark.

### SFGS subscale outcomes

SFGS subscale trajectories are shown in Supplementary Table S1. Voluntary movement scores improved in both groups but increased more in the intervention group at each follow-up time point. At Week 12, voluntary movement score was 85.4 ± 11.6 in the intervention group and 75.5 ± 12.0 in the control group, with an adjusted between-group difference of 9.7 points (95% CI, 5.7–13.6; *p* < 0.001).

Resting symmetry scores decreased over time in both groups, indicating improvement in resting facial asymmetry. At Week 12, resting asymmetry score was 1.0 ± 2.0 in the intervention group and 3.6 ± 2.3 in the control group, with an adjusted between-group difference of −2.6 points (95% CI, −3.3 to −1.9; *p* < 0.001). Synkinesis scores remained low overall, although Week 12 synkinesis scores were lower in the intervention group than in the control group, with mean scores of 1.9 ± 0.4 and 2.3 ± 0.5, respectively, and an adjusted between-group difference of −0.5 points (95% CI, −0.6 to −0.3; *p* < 0.001).

### Secondary patient-reported and symptom outcomes

Secondary patient-reported and symptom outcomes from baseline to Week 12 are summarized in Table 3. FDI physical function improved from 62.2 ± 21.1 to 85.2 ± 15.6 in the intervention group, compared with 61.6 ± 17.9–62.0 ± 23.8 in the control group. The adjusted between-group difference at Week 12 was 23.1 points (95% CI, 16.5–29.7; *p* < 0.001). FDI social/wellbeing score also improved more in the intervention group, with an adjusted between-group difference of 9.2 points (95% CI, 3.0–15.3; *p* = 0.003).

**Table 3:** Secondary patient-reported and symptom outcomes from baseline to Week 12.

Outcome	Baseline intervention (*n* = 84)	Baseline control (*n* = 76)	Week 12 intervention (*n* = 84)	Week 12 control (*n* = 76)	Change intervention	Change control	Adjusted between-group difference (95% CI)	*p* value
FDI physical function score	62.2 ± 21.1	61.6 ± 17.9	85.2 ± 15.6	62.0 ± 23.8	23.0 ± 25.7	0.4 ± 31.0	23.1 (16.5–29.7)	<0.001
FDI social/wellbeing score	49.5 ± 16.4	51.6 ± 16.3	67.0 ± 19.6	58.1 ± 18.0	17.5 ± 25.2	6.4 ± 23.4	9.2 (3.0–15.3)	0.003
FaCE total score	48.1 ± 10.8	48.5 ± 15.5	76.0 ± 20.3	67.9 ± 21.9	27.9 ± 21.4	19.4 ± 27.3	7.9 (0.8–14.9)	0.028
Pain NRS score	3.1 ± 0.8	2.9 ± 0.9	0.0 ± 0.1	0.2 ± 0.4	−3.1 ± 0.8	−2.7 ± 1.0	−0.2 (−0.3 to −0.1)	<0.001
Stiffness NRS score	4.3 ± 1.1	4.0 ± 1.0	2.8 ± 0.6	3.9 ± 0.7	−1.5 ± 1.3	−0.2 ± 1.3	−1.1 (−1.3 to −0.9)	<0.001

Values are mean ± SD unless otherwise indicated. Changes were calculated as Week 12 minus baseline; positive changes indicate improvement for FDI and FaCE scores, whereas negative changes indicate improvement for pain and stiffness NRS scores. Adjusted between-group differences are intervention minus control and were estimated using separate multivariable linear regression models for each Week 12 outcome, adjusted for the corresponding baseline outcome, time from symptom onset to baseline, baseline House–Brackmann grade, age, sex, and diabetes. Heteroscedasticity-consistent standard errors were used. *p* values correspond to the adjusted between-group differences. Higher FDI and FaCE scores indicate better function or quality of life; pain and stiffness were assessed using 0–10 numeric rating scales, with higher scores indicating greater symptom severity. CI, confidence interval; FaCE, Facial Clinimetric Evaluation; FDI, Facial Disability Index; HB, House-Brackmann; NRS, numeric rating scale; SD, standard deviation.

FaCE total score increased from 48.1 ± 10.8 to 76.0 ± 20.3 in the intervention group and from 48.5 ± 15.5 to 67.9 ± 21.9 in the control group. The adjusted between-group difference was 7.9 points (95% CI, 0.8–14.9; *p* = 0.028). Pain scores decreased substantially in both groups, reaching 0.0 ± 0.1 in the intervention group and 0.2 ± 0.4 in the control group at Week 12; the adjusted between-group difference was −0.2 points (95% CI, −0.3 to −0.1; *p* < 0.001). Stiffness score decreased from 4.3 ± 1.1 to 2.8 ± 0.6 in the intervention group and from 4.0 ± 1.0 to 3.9 ± 0.7 in the control group, with an adjusted between-group difference of −1.1 points (95% CI, −1.3 to −0.9; *p* < 0.001).

Although the adjusted pain difference was statistically significant, its magnitude was only 0.2 points on the 0–10 NRS and was considered clinically negligible. No established facial-palsy-specific threshold was available for interpreting the clinical importance of the stiffness NRS difference; therefore, its magnitude is reported without assigning a definitive clinically important effect.

### House–Brackmann outcomes

House–Brackmann outcomes at Week 12 are presented in Table 4. Mean Week 12 HB grade was 2.1 ± 0.6 in the intervention group and 2.7 ± 0.5 in the control group, with an adjusted mean difference of −0.56 grade units (95% CI, −0.73 to −0.39; *p* < 0.001). Mean HB grade improvement from baseline to Week 12 was 1.9 ± 0.9 grades in the intervention group and 1.4 ± 0.8 grades in the control group (mean difference, 0.56; 95% CI, 0.31–0.81; *p* < 0.001).

**Table 4:** House–Brackmann grade outcomes at Week 12.

Outcome	Intervention (*n* = 84)	Control (*n* = 76)	Effect estimate (95% CI)	*p* value
Baseline HB grade	4.0 ± 0.6	4.0 ± 0.6	MD − 0.01 (−0.21 to 0.18)	0.892
Week 12 HB grade	2.1 ± 0.6	2.7 ± 0.5	Adjusted MD − 0.56 (−0.73 to −0.39)	<0.001
HB grade improvement	1.9 ± 0.9	1.4 ± 0.8	MD 0.56 (0.31–0.81)	<0.001
≥1-grade HB improvement	83 (98.8)	69 (90.8)	RD 8.0 pp. (1.5–17.2)	0.028
≥2-grade HB improvement	59 (70.2)	29 (38.2)	RD 32.1 pp. (16.8–45.8)	<0.001
HB grade I–II at Week 12	67 (79.8)	26 (34.2)	RD 45.6 pp. (30.8–58.1)	<0.001

Values are mean ± SD or *n* (%), as indicated. House–Brackmann grade improvement was calculated as baseline grade minus Week 12 grade; positive values indicate clinical improvement. For continuous House–Brackmann outcomes, mean differences are intervention minus control; the *p* values for baseline grade and grade improvement were calculated using Mann–Whitney *U* tests because House–Brackmann grade is ordinal. The adjusted mean difference for Week 12 House–Brackmann grade was estimated using linear regression adjusted for baseline House–Brackmann grade, baseline Sunnybrook total score, time from symptom onset to baseline, age, sex, and diabetes, with heteroscedasticity-consistent standard errors. For binary outcomes, between-group estimates are absolute risk differences in percentage points, calculated as intervention minus control, with score 95% confidence intervals; *p* values were calculated using Fisher’s exact test. Lower House–Brackmann grades indicate better facial nerve function. CI, confidence interval; HB, House–Brackmann; MD, mean difference; pp, percentage points; RD, risk difference; SD, standard deviation.

A ≥ 1-grade HB improvement occurred in 83 patients (98.8%) in the intervention group and 69 patients (90.8%) in the control group, corresponding to a risk difference of 8.0 percentage points (95% CI, 1.5–17.2; *p* = 0.028). A ≥ 2-grade improvement was observed in 59 patients (70.2%) in the intervention group and 29 patients (38.2%) in the control group, with a risk difference of 32.1 percentage points (95% CI, 16.8–45.8; *p* < 0.001). At Week 12, 67 patients (79.8%) in the intervention group achieved HB grade I–II compared with 26 patients (34.2%) in the control group, corresponding to a risk difference of 45.6 percentage points (95% CI, 30.8–58.1; *p* < 0.001). The full ordinal distribution of HB grades at baseline and Week 12 is provided in Supplementary Table S2.

### Exploratory subgroup analyses

Exploratory subgroup analyses showed directionally favorable adjusted between-group differences in Week 12 SFGS total score among participants receiving the multicomponent rehabilitation program across the examined subgroups (Table 5). The adjusted between-group difference was 19.2 points (95% CI, 2.6–35.7; *p* = 0.023) among patients enrolled within 7 days of symptom onset and 12.5 points (95% CI, 8.3–16.7; *p* < 0.001) among those enrolled 8–14 days after onset, with no evidence of interaction by onset timing (*p* for interaction = 0.596). The between-group difference was also consistent across baseline severity, age, sex, and diagnosis subgroups, with all interaction *p* values >0.05.

**Table 5:** Exploratory subgroup analysis of the Week 12 Sunnybrook Facial Grading System total score.

Subgroup	Level	Intervention *n*	Control *n*	Week 12 intervention	Week 12 control	Adjusted between-group difference (95% CI)	*p* value	*p* for interaction
Overall cohort	All participants	84	76	82.5 ± 11.8	69.5 ± 11.5	12.7 (8.8–16.6)	<0.001	–
Onset-to-baseline	≤7 days	9	7	80.9 ± 10.3	65.0 ± 5.9	19.2 (2.6–35.7)	0.023	0.596
Onset-to-baseline	8–14 days	75	69	82.7 ± 12.1	70.0 ± 11.9	12.5 (8.3–16.7)	<0.001	
Baseline severity	HB III–IV	74	68	82.0 ± 11.7	69.6 ± 11.8	12.2 (8.0–16.3)	<0.001	0.366
Baseline severity	HB V–VI	10	8	86.3 ± 12.5	68.6 ± 9.2	15.5 (2.7–28.3)	0.018	
Age	<45 years	37	39	81.2 ± 12.9	71.1 ± 12.2	9.4 (3.0–15.8)	0.004	0.123
Age	≥45 years	47	37	83.6 ± 10.9	67.9 ± 10.7	15.7 (10.4–20.9)	<0.001	
Sex	Female	41	32	82.0 ± 10.9	70.4 ± 12.3	11.2 (5.4–17.0)	<0.001	0.526
Sex	Male	43	44	83.0 ± 12.8	68.8 ± 11.0	13.9 (7.8–19.9)	<0.001	
Diagnosis	Bell palsy	64	64	82.3 ± 11.2	70.0 ± 11.4	11.9 (7.7–16.1)	<0.001	0.438
Diagnosis	Post-infectious	20	12	83.2 ± 13.9	67.1 ± 12.5	15.1 (3.1–27.0)	0.013	

Values are mean ± SD unless otherwise indicated. The primary outcome in this exploratory table is the Week 12 Sunnybrook Facial Grading System total score. Adjusted between-group differences are intervention minus control; positive values favor the intervention. The overall cohort estimate corresponds to the primary adjusted model. Within each subgroup level, adjusted differences were estimated using linear regression adjusted for baseline Sunnybrook total score, time from symptom onset to baseline, baseline House–Brackmann grade, age, sex, and diabetes, excluding the subgroup-defining variable when applicable. Heteroscedasticity-consistent standard errors were used. Interaction *p* values were estimated from models including the treatment group, subgroup indicator, group-by-subgroup interaction term, and the same covariates. Subgroup analyses were prespecified as exploratory, were not adjusted for multiplicity, and should be interpreted as assessing consistency rather than definitive treatment-effect modification. CI, confidence interval; HB, House–Brackmann; SD, standard deviation; SFGS, Sunnybrook Facial Grading System.

### Sensitivity analyses

Sensitivity analyses yielded similar estimates for the primary Week 12 SFGS between-group difference across alternative analytic specifications (Table 6). The unadjusted Week 12 score difference was 13.0 points (95% CI, 9.4–16.6; *p* < 0.001), and the unadjusted change-score comparison showed a between-group difference of 13.5 points (95% CI, 9.6–17.5; *p* < 0.001). Further analysis produced between-group estimates ranging from 12.7 to 13.5 Sunnybrook points, with all *p* values <0.001. The similarity of these should not be interpreted as evidence that residual or unmeasured bias was eliminated.

**Table 6:** Sensitivity analyses for the Week 12 Sunnybrook Facial Grading System between-group difference.

Sensitivity analysis	Outcome	Model specification	Between-group difference (95% CI)	*p* value
Unadjusted Week 12 score difference	Week 12 SFGS total score	Treatment group only	13.0 (9.4–16.6)	<0.001
Change-score comparison	Change in SFGS total score from baseline to Week 12	Treatment group only	13.5 (9.6–17.5)	<0.001
Baseline-adjusted ANCOVA	Week 12 SFGS total score	Baseline SFGS total score	12.9 (9.3–16.6)	<0.001
Parsimonious adjusted model	Week 12 SFGS total score	Baseline SFGS total score, onset-to-baseline time, and baseline HB grade	12.9 (9.1–16.6)	<0.001
Primary adjusted model	Week 12 SFGS total score	Baseline SFGS total score, onset-to-baseline time, baseline HB grade, age, sex, and diabetes	12.7 (8.8–16.6)	<0.001
Expanded covariate model	Week 12 SFGS total score	Primary model plus steroid start day, diagnosis type, and hypertension	12.7 (8.7–16.7)	<0.001
Propensity-score weighted analysis	Week 12 SFGS total score	Stabilized inverse-probability weighting based on baseline covariates	12.7 (8.9–16.6)	<0.001
Robust regression	Week 12 SFGS total score	Huber M-estimation with the primary covariate set	13.2 (9.2–17.2)	<0.001
Median regression	Median Week 12 SFGS total score	Quantile regression with the primary covariate set	13.4 (8.1–18.8)	<0.001
Repeated-measures GEE	Week 12 treatment contrast across the SFGS trajectory	Group, time, group-by-time interaction, and prespecified covariates	12.9 (9.3–16.5)	<0.001

Effects are reported as intervention minus control in points on the Sunnybrook Facial Grading System total score unless otherwise indicated; positive values favor the intervention. All analyses used complete observed data because no missing values were present in the cleaned dataset. Linear regression models used heteroscedasticity-consistent standard errors. The primary adjusted model corresponds to the main Week 12 analysis. The expanded model additionally included steroid start day from symptom onset, diagnosis type, and hypertension. The propensity-score analysis used stabilized inverse-probability weights estimated from baseline SFGS total score, onset-to-baseline time, baseline HB grade, age, sex, diabetes, steroid start day, diagnosis type, and hypertension. The repeated-measures GEE model included all SFGS time points from baseline to Week 12 with an exchangeable working correlation structure; the global group-by-time interaction was significant (*p* < 0.001). ANCOVA, analysis of covariance; CI, confidence interval; GEE, generalized estimating equation; HB, House–Brackmann; SFGS, Sunnybrook Facial Grading System.

## Discussion

In this prospective controlled cohort of adults with acute or early-subacute unilateral peripheral facial nerve palsy, participants who received usual care plus the standardized multicomponent rehabilitation program had more favorable short-term facial-function and patient-reported outcomes than participants receiving usual care alone. The adjusted Week 12 difference in SFGS total score was approximately 13 points, and directionally similar between-group differences were observed across several secondary outcomes and analytic specifications. These findings should, however, be interpreted as associations with receipt of the combined program rather than evidence that the program caused better recovery. Allocation was not randomized and was influenced by clinician recommendation and patient preference, outcome assessors were not blinded, and the usual-care group did not receive an attention-matched intervention. Residual confounding, treatment preference, expectation or contextual effects, differential therapeutic attention, motivation, and other unmeasured factors may therefore have contributed to the observed differences. Furthermore, because the program combined acupuncture, massage, supervised motor retraining, and home exercise, the present study cannot attribute the observed association specifically to acupuncture or to any other individual component.

The distinction between statistical significance and clinical importance is particularly important when interpreting the observed recovery trajectory, given the substantial spontaneous improvement expected in acute peripheral facial palsy. Using an externally supported 10-point SFGS benchmark, the adjusted between-group difference at Week 2 (7.5 points) remained below this threshold, whereas the Week 4 difference (15.3 points) exceeded it across the entire confidence interval. At Weeks 8 and 12, the point estimates also exceeded 10 points, although their confidence intervals included smaller differences; these findings are therefore compatible with, but do not conclusively establish, a clinically important between-group difference. Both groups improved substantially over time, as expected from the natural history of acute facial palsy, while greater separation between groups was already observed by Week 2 and became more pronounced by Week 4. This temporal pattern suggests an association between receipt of the combined rehabilitation program and more favorable early outcomes, but it should not be interpreted as evidence that the program itself accelerated recovery because allocation was non-randomized and spontaneous recovery, residual confounding, and other nonspecific influences cannot be excluded. The observed pattern is nevertheless consistent with previous rehabilitation studies suggesting that structured facial exercise or retraining may be beneficial when introduced early and systematically (6, 7), including a recent randomized trial in which facial stretching combined with structured exercise produced greater short-term improvement in SFGS scores than conventional exercise in acute Bell’s palsy (10).

Our results are also consistent with prior work suggesting that early rehabilitation may be particularly relevant in patients with more severe facial weakness. Nicastri and colleagues reported that early physical therapy appeared most beneficial among patients with severe Bell’s palsy, especially those with House–Brackmann grade V–VI at baseline, whereas less severe cases showed substantial spontaneous recovery (8). In the present study, the adjusted between-group difference was directionally favorable among participants receiving the combined program in both HB III–IV and HB V–VI subgroups, although the number of patients with severe baseline palsy was small. Therefore, these subgroup results should be interpreted as evidence of consistency with previous findings. Similarly, the Mirror Effect Plus Protocol trial suggested that structured mirror-based rehabilitation initiated during the acute phase may improve longer-term facial recovery in patients with moderately severe to total Bell’s palsy (9). Together with prior randomized studies, these observations provide a rationale for further evaluation of early, standardized, supervised rehabilitation, but the present observational data do not establish superiority or efficacy of the combined program.

The SFGS subscale findings provide further insight into the pattern of recovery. The intervention group showed greater improvement in voluntary movement and resting facial symmetry, while synkinesis scores remained low overall. This is expected in an acute or early-subacute cohort because clinically meaningful synkinesis often develops later, after aberrant regeneration and maladaptive motor patterns become established. Contemporary reviews of facial synkinesis emphasize that synkinesis is typically a delayed sequela and that multidimensional facial grading is preferable to relying solely on global scales (19). Week 12 is an appropriate endpoint for early functional recovery, but it is likely early to determine whether the intervention reduces late synkinesis, contracture, or persistent maladaptive co-contraction. Longer follow-up, ideally at 6 and 12 months, would be required to address this question.

The House–Brackmann findings provide an additional clinically interpretable description of recovery, although a mean difference in an ordinal grade should not itself be treated as an MCID. More informative clinically are the categorical recovery thresholds: at Week 12, 79.8% of participants receiving the combined program versus 34.2% receiving usual care reached Grade I–II, and 70.2% versus 38.2% achieved an improvement of at least two grades. These substantial absolute differences provide descriptive clinical context, although, given the non-randomized design, they represent associations rather than causal treatment effects. Prior comparison of SFGS and House–Brackmann grading has shown that the two systems correlate but do not capture facial function identically; SFGS may be more responsive to regional changes in facial function and recovery over time (20).

Patient-reported outcomes showed parallel between-group differences in several patient-centered domains. The intervention group showed larger gains in FDI physical function, FDI social/wellbeing, and FaCE total score. This is important because facial palsy affects more than visible facial movement. Patients may experience difficulty eating, drinking, speaking, expressing emotion, maintaining eye comfort, and participating confidently in social interactions. The FDI and FaCE scales were developed to capture these patient-centered dimensions of facial neuromuscular dysfunction and facial-palsy-specific quality of life (16, 17). In the present study, the statistical differences in FDI physical function indicate a larger observed improvement in patient-reported physical function among participants receiving the combined program. The smaller but significant improvements in FDI social/wellbeing and FaCE total score suggest broader patient-perceived recovery beyond clinician-rated motor improvement.

The symptom findings also offer practical clinical insight. Pain improved markedly in both groups and was nearly absent by Week 12, with only a small adjusted between-group difference. This is consistent with the clinical course of acute Bell’s palsy, in which pain is often most relevant early and tends to decrease over time. By contrast, stiffness showed a more statistically significant between-group difference. Facial stiffness and tightness are commonly reported symptoms in patients recovering from facial palsy and may persist even when gross movement improves. Previous work on facial rehabilitation, including mime therapy, has shown improvement in stiffness, lip mobility, and social and physical aspects of facial disability in patients with facial paresis (21, 22) Our findings extend this concept to an acute or early-subacute controlled cohort and suggest that stiffness may be a useful patient-centered secondary outcome in future facial palsy rehabilitation studies.

The pattern of patient-reported recovery in the usual-care group warrants specific consideration. Despite substantial improvement in clinician-rated SFGS and House–Brackmann outcomes, mean FDI physical function changed only minimally, from 61.6 at baseline to 62.0 at Week 12. This apparent discordance should not necessarily be interpreted as contradictory because clinician-rated facial grading and patient-reported physical disability capture related but distinct aspects of recovery. The SFGS and House–Brackmann systems primarily characterize observable facial motor function, whereas the FDI physical domain reflects patients’ perceived ability to perform everyday facial functions. Previous psychometric evidence has likewise shown only a modest association between SFGS and FDI physical-function scores (23). Moreover, the limited mean change in FDI physical function did not represent an absence of improvement across all patient-reported measures in the usual-care group: FDI social/wellbeing and FaCE total scores both improved over follow-up. Individual changes in FDI physical function were also highly heterogeneous, indicating that the near-zero group mean may mask substantial variability in patient-level trajectories. Taken together, these findings suggest that recovery in clinician-observed facial movement did not translate uniformly into perceived physical functioning in the usual-care group. The present data cannot determine whether this divergence reflects persistence of activity-level limitations, differences in measurement constructs or responsiveness, or other patient-level factors, and the finding should therefore be interpreted cautiously.

The present study adds to the literature in several ways. First, it evaluated a combined and standardized acupuncture-assisted facial massage and exercise protocol rather than a loosely defined or single-component rehabilitation approach. Second, it followed patients serially from baseline through Week 12, allowing assessment of recovery trajectory rather than relying only on a single endpoint. Third, it integrated clinician-rated facial grading, patient-reported disability and quality-of-life measures, and symptom outcomes. Fourth, the estimated between-group difference was numerically similar across multiple analytic approaches, including baseline-adjusted models, expanded covariate adjustment, propensity-score weighting, robust regression, median regression, and repeated-measures GEE. This consistency indicates that the magnitude and direction of the observed association were relatively stable across the examined model specifications. It does not, however, demonstrate freedom from bias or strengthen causal inference, because these analyses cannot account for unmeasured confounding or nonspecific influences associated with non-randomized treatment selection.

The results also need to be interpreted in light of important limitations. The most important limitation is the observational, non-randomized design. Although the study was analyzed as a controlled cohort and included repeated follow-up assessments, treatment allocation was not randomized. Therefore, the findings should be interpreted as associations between receiving the standardized protocol and improved recovery, not as definitive evidence of direct causality. Baseline characteristics were broadly comparable, and the analyses adjusted for clinically important confounders, including baseline facial function, time from onset, baseline HB grade, age, sex, and diabetes. However, residual confounding remains possible. Patients who received the intervention may have differed from controls in motivation, expectations, access to care, clinician attention, adherence to home exercises, or other unmeasured factors that could influence recovery.

Several additional limitations should be acknowledged. The principal limitation of this study is its observational, non-randomized design. Allocation was influenced by clinician recommendation and patient preference, creating the possibility of confounding by treatment preference, motivation, access to care, and other measured or unmeasured factors. Although baseline characteristics were broadly comparable and several analytic approaches were used to account for measured confounders, these methods cannot establish exchangeability between groups or eliminate residual confounding. An additional limitation is the absence of blinded outcome assessment. Clinician-rated outcomes, including the SFGS and House–Brackmann grade, were assessed by a single researcher who was aware of group assignment, and these measures involve an element of clinical judgment. Although use of the same assessor throughout the study reduced variability arising from differences between raters, it does not eliminate the possibility of observer bias. Because only one assessor was used, inter-rater reliability could not be estimated, and standardized facial photographs or videos were not obtained for retrospective blinded reassessment. Consequently, the magnitude of potential observer-related measurement bias cannot be quantified and should be considered when interpreting the clinician-rated between-group differences. Additionally, the program itself was multicomponent, combining facial acupuncture, therapist-assisted facial massage, supervised facial movement training, and home exercise. The study therefore cannot determine the independent contribution of any component and, in particular, cannot be interpreted as evidence for an acupuncture-specific effect. Moreover, the follow-up duration was limited to 12 weeks, which is appropriate for early recovery but insufficient for definitive assessment of late synkinesis, contracture, durability of benefit, or recurrence. The control group received usual care, but the intensity and consistency of general facial advice, unsupervised exercise, eye care, analgesic use, and other supportive measures may not have been fully captured. Detailed adherence and safety variables, such as session attendance, home-exercise completion, adverse events, and analgesic use, were not available in our study. Moreover, the usual-care group was not attention matched. Participants receiving the combined program had repeated structured contact with a treating practitioner in addition to acupuncture, massage, supervised exercise, and home training. Consequently, expectation, placebo or other contextual effects, differential therapeutic attention, reinforcement of adherence, and therapeutic interaction may have contributed to the observed between-group differences. Neither conventional covariate adjustment nor propensity-score weighting can remove these influences when they were not measured or were intrinsic to the treatment context. The sensitivity analyses should therefore be interpreted as demonstrating stability across the examined statistical specifications rather than as evidence that the principal sources of observational bias were eliminated. Finally, because the cohort focused on adults with acute or early-subacute unilateral peripheral facial nerve palsy, the findings may not generalize to recurrent palsy, bilateral palsy, traumatic or postsurgical facial nerve injury, Ramsay Hunt syndrome, or chronic synkinesis-dominant presentations.

Given these limitations, the present findings should not be interpreted as evidence that the combined program is an efficacy-proven treatment or that acupuncture itself improves facial palsy recovery. Rather, the study provides preliminary, hypothesis-generating evidence that receipt of a standardized multicomponent rehabilitation program was associated with more favorable short-term outcomes than usual care in this cohort. These observations provide a rationale for prospective randomized evaluation but are not sufficient to establish clinical effectiveness or to recommend any individual component of the program on the basis of the present study alone.

Future studies should test these hypothesis-generating observations in randomized controlled trials with concealed allocation, blinded outcome assessment, standardized usual care, and an appropriately designed attention-matched comparator. Future trials should also record intervention fidelity, session attendance, home-exercise adherence, adverse events, analgesic use, patient expectations, and co-interventions. Longer follow-up at 6 and 12 months would be particularly important to determine whether early structured rehabilitation reduces late synkinesis, residual stiffness, or long-term facial disability. Because telehealth and home-based rehabilitation are increasingly feasible, future studies could also compare supervised in-person protocols with hybrid or telerehabilitation-supported facial exercise programs (24). Finally, factorial, dismantling, or appropriately controlled component-specific designs would additionally be required to distinguish any acupuncture-specific effect from the contributions of massage, supervised motor retraining, home exercise, therapeutic attention, expectation, and other contextual effects.

## Conclusion

This prospective controlled cohort study found that receipt of a standardized multicomponent facial rehabilitation program comprising facial acupuncture, therapist-assisted massage, supervised facial movement training, and home exercise, in addition to usual care, was associated with more favorable short-term clinician-rated and patient-reported outcomes over 12 weeks. Because allocation was non-randomized and preference based, outcome assessors were not blinded, and the comparator was not attention matched, the observed differences cannot establish causality and may partly reflect residual confounding, expectation or contextual effects, and differential therapeutic attention. The multicomponent design also precludes attributing the observed association specifically to acupuncture. These hypothesis-generating findings require confirmation in randomized trials with concealed allocation, blinded outcome assessment, and appropriate comparator conditions.

## Data Availability

The raw data supporting the conclusions of this article will be made available by the authors, without undue reservation.
