# Supplementary material for: Short-term clinical outcomes associated with a standardized multicomponent facial rehabilitation program in acute or early-subacute peripheral facial nerve palsy: a prospective controlled cohort study
Source: Front Neurol. 2026 Sep 23;17:1931200. doi: 10.3389/fneur.2026.1931200 (PMC13642283; doi:10.3389/fneur.2026.1931200)
Supplement: Supplementary file 1 [file Table_1.DOCX]

**Supplementary Table S1. Sunnybrook Facial Grading System subscale trajectories from baseline to Week 12**

| **Sunnybrook domain** | **Time point** | **Intervention (n = 84)** | **Control (n = 76)** | **Intervention change from baseline** | **Control change from baseline** | **Adjusted between-group difference (95% CI)** | **p value** |
| --- | --- | --- | --- | --- | --- | --- | --- |
| Voluntary movement | Baseline | 51.3 ± 3.7 | 51.8 ± 4.3 | — | — | — | 0.384 |
|  | Week 2 | 62.2 ± 5.6 | 57.1 ± 6.0 | 10.9 ± 7.1 | 5.2 ± 7.5 | 5.0 (3.1 to 6.9) | <0.001 |
|  | Week 4 | 70.9 ± 8.8 | 64.1 ± 10.2 | 19.6 ± 10.0 | 12.2 ± 11.2 | 6.9 (3.8 to 10.0) | <0.001 |
|  | Week 8 | 78.9 ± 13.6 | 69.5 ± 13.9 | 27.6 ± 13.8 | 17.6 ± 13.8 | 9.1 (4.7 to 13.5) | <0.001 |
|  | Week 12 | 85.4 ± 11.6 | 75.5 ± 12.0 | 34.1 ± 12.3 | 23.6 ± 12.7 | 9.7 (5.7 to 13.6) | <0.001 |
| Resting symmetry | Baseline | 15.2 ± 0.9 | 15.1 ± 0.6 | — | — | — | 0.354 |
|  | Week 2 | 9.9 ± 0.8 | 12.3 ± 2.5 | -5.3 ± 1.2 | -2.8 ± 2.5 | -2.4 (-3.0 to -1.8) | <0.001 |
|  | Week 4 | 0.9 ± 1.9 | 9.2 ± 1.8 | -14.3 ± 2.2 | -5.9 ± 1.9 | -8.3 (-8.9 to -7.7) | <0.001 |
|  | Week 8 | 0.9 ± 1.9 | 3.7 ± 2.2 | -14.3 ± 2.2 | -11.4 ± 2.3 | -2.8 (-3.5 to -2.2) | <0.001 |
|  | Week 12 | 1.0 ± 2.0 | 3.6 ± 2.3 | -14.2 ± 2.2 | -11.4 ± 2.3 | -2.6 (-3.3 to -1.9) | <0.001 |
| Synkinesis | Baseline | 0.3 ± 0.5 | 0.5 ± 0.6 | — | — | — | 0.134 |
|  | Week 2 | 1.0 ± 0.2 | 1.0 ± 0.2 | 0.6 ± 0.5 | 0.6 ± 0.6 | -0.0 (-0.1 to 0.0) | 0.160 |
|  | Week 4 | 1.1 ± 0.4 | 1.4 ± 0.5 | 0.8 ± 0.7 | 0.9 ± 0.8 | -0.3 (-0.4 to -0.2) | <0.001 |
|  | Week 8 | 1.4 ± 0.5 | 1.9 ± 0.4 | 1.1 ± 0.7 | 1.4 ± 0.7 | -0.4 (-0.6 to -0.3) | <0.001 |
|  | Week 12 | 1.9 ± 0.4 | 2.3 ± 0.5 | 1.5 ± 0.7 | 1.9 ± 0.8 | -0.5 (-0.6 to -0.3) | <0.001 |

Values are mean ± SD unless otherwise indicated. Changes were calculated as follow-up score minus baseline score. Adjusted between-group differences are intervention minus control and were estimated using separate linear regression models for each follow-up subscale, adjusted for the corresponding baseline subscale, time from symptom onset to baseline, baseline House–Brackmann grade, age, sex, and diabetes. Heteroscedasticity-consistent standard errors were used. Baseline p values are descriptive and were calculated using Welch’s independent-samples t test for voluntary movement and resting symmetry and the Mann–Whitney U test for synkinesis. Higher voluntary movement scores indicate better function, whereas lower resting symmetry and synkinesis scores indicate better status. Therefore, positive between-group differences favor the intervention for voluntary movement, and negative between-group differences favor the intervention for resting symmetry and synkinesis. **Abbreviations**: CI, confidence interval; HB, House–Brackmann; SD, standard deviation; SFGS, Sunnybrook Facial Grading System.

**Supplementary Table S2. Distribution of House–Brackmann grades at baseline and Week 12**

| **Time point** | **House-Brackmann grade** | **Intervention (n = 84)** | **Control (n = 76)** | **p value** |
| --- | --- | --- | --- | --- |
| Baseline | Grade I | 0 (0.0) | 0 (0.0) | 0.892 |
|  | Grade II | 0 (0.0) | 0 (0.0) |  |
|  | Grade III | 12 (14.3) | 11 (14.5) |  |
|  | Grade IV | 62 (73.8) | 57 (75.0) |  |
|  | Grade V | 8 (9.5) | 4 (5.3) |  |
|  | Grade VI | 2 (2.4) | 4 (5.3) |  |
| Week 12 | Grade I | 10 (11.9) | 0 (0.0) | <0.001 |
|  | Grade II | 57 (67.9) | 26 (34.2) |  |
|  | Grade III | 17 (20.2) | 50 (65.8) |  |
|  | Grade IV | 0 (0.0) | 0 (0.0) |  |
|  | Grade V | 0 (0.0) | 0 (0.0) |  |
|  | Grade VI | 0 (0.0) | 0 (0.0) |  |

Values are n (%). p values compare the ordinal House–Brackmann grade distribution between treatment groups at each time point using the Mann–Whitney U test. Lower House–Brackmann grades indicate better facial nerve function. The same Week 12 distribution is summarized clinically in the main manuscript table using mean grade, grade improvement, and recovery thresholds. **Abbreviations**: HB, House–Brackmann.
